# *Escherichia coli* in Brazilian Poultry Fecal Samples: Co-Carriage of Fosfomycin and ESBL Resistance

**DOI:** 10.3390/antibiotics14030269

**Published:** 2025-03-06

**Authors:** Felipe Juscele, Andre B. S. Saidenberg, Lars E. B. Christoffersen, Sofie M. Edslev, Søren Hallstrøm, Jessica R. Nacarato, Fernanda B. Barbosa, Marcos P. Cunha, Fernanda Esposito, Nilton H. Lincopan, Marc Stegger, Terezinha Knöbl

**Affiliations:** 1 School of Veterinary Medicine and Animal Science, Universidade de São Paulo, São Paulo 05508-270, Brazil; 2 Department of Sequencing and Bioinformatics, Statens Serum Institut, 2300 Copenhagen, Denmark; 3 Department of Microbiology, Institute of Biomedical Sciences, Universidade de São Paulo, São Paulo 12247-016, Brazil; 4 Antimicrobial Resistance and Infectious Diseases Laboratory, Harry Butler Institute, Murdoch University, Murdoch, WA 6150, Australia

**Keywords:** *Escherichia coli*, fosfomycin, poultry

## Abstract

**Background/Objectives**: Fosfomycin, a critically important antibiotic, is widely used to treat urinary tract infections (UTIs) caused by multidrug-resistant (MDR) *Escherichia coli*, particularly those producing extended-spectrum β-lactamases (ESBLs). However, its increasing use in livestock has raised concerns about resistance development and global dissemination. This study investigated fosfomycin resistance in *E. coli* isolates from 400 fecal samples collected at Brazilian broiler farms. **Methods**: The samples were tested for their minimum inhibitory concentration (MIC), screened with PCR for specific resistance genes, and selected isolated were whole genome sequenced. **Results**: Phenotypic resistance to fosfomycin was detected in 19% (75/400) of the isolates, while the *fosA3* gene, encoding enzymatic resistance, was identified in 4% (16/400) via PCR screening. Long-read sequencing of seven *fosA3*-positive isolates revealed the presence of *fosA3* on IncFII and IncX plasmids, often co-located with *bla*_CTX-M-55_ within a conserved *IS*26-flanked transposon. Comparative genomic analysis of 133,541 global *E. coli* genomes from EnteroBase showed that 35% harbored similar transposon structures, with 2% carrying *fosA3*. These *fosA3*-positive isolates were significantly associated with South America and exhibited high co-carriage of ESBL genes, particularly in environmental and poultry-associated isolates. Phylogenetic analysis demonstrated no clustering by host or geographic origin, highlighting the global dissemination of these resistance determinants. **Conclusions**: Our findings emphasize the role of poultry production in the spread of fosfomycin and ESBL resistance, driven by transmissible plasmids and co-selection with third-generation cephalosporins. Improved antimicrobial stewardship, surveillance programs, and alternative management strategies are urgently needed to mitigate the dissemination of resistance and preserve fosfomycin’s efficacy in human medicine.

## 1. Introduction

Fosfomycin is a broad-spectrum antibiotic with a unique mechanism of action that inhibits bacterial cell wall synthesis through inactivation of the MurA enzyme, a pathway distinct from other antimicrobial classes [1]. Due to this mechanism, fosfomycin exhibits limited cross-resistance with other antibiotics, making it a critical treatment option for multidrug-resistant (MDR) and extensively drug-resistant (XDR) infections. Fosfomycin is a critically important antimicrobial for human medicine, a first-line treatment for uncomplicated urinary tract infections (UTIs), and is increasingly utilized in combination therapies to treat infections caused by extended-spectrum β-lactamase (ESBL)-producing *Escherichia coli* and other MDR pathogens [2].

The growing global use of fosfomycin, both in human and veterinary medicine, has raised significant concerns regarding the emergence of resistance [3]. Fosfomycin resistance mechanisms can arise through chromosomal mutations, such as modifications to the MurA enzyme or reduced membrane permeability, as well as through the acquisition of plasmid-borne resistance genes [1,4]. Among these, the *fosA3* gene has emerged as a major contributor to fosfomycin resistance, encoding enzymes that inactivate the antibiotic [5]. The *fosA3* gene is often co-located with other resistance determinants, such as *bla_CTX-M_* types, on highly transmissible plasmids, further complicating treatment strategies [3]. Previous studies have reported the presence of *fosA3*-positive *E. coli* in poultry farms, retail meat, and environmental samples in Brazil, underscoring the contribution of agricultural practices to the AMR (antimicrobial resistance) burden [6,7].

Brazil, as one of the largest poultry producers and the leading global exporter of poultry meat [8], plays a critical role in the spread of AMR. Intensive poultry production systems in Brazil rely on antimicrobial agents to manage frequent bacterial infections [9], such as colibacillosis, a disease caused by avian pathogenic *E. coli* [10].

Most research on fosfomycin resistance has been performed in Asian countries, where resistance to this drug is very prevalent [10,11,12], and despite its increasing critical importance in human medicine worldwide [13,14,15,16], there is a shortage of genomic studies concerning the genetic context and dissemination of *fosA* genes in South America [17], where several countries allow the use of fosfomycin to treat livestock.

This study aimed to evaluate the frequency of phenotypic and genotypic fosfomycin resistance in *E. coli* isolates from fecal samples collected at Brazilian broiler farms. Whole-genome sequencing (WGS) was performed on selected resistant isolates to characterize the genetic structures surrounding *fosA3* and to identify associations with ESBL genes. Comparative genomic analyses with global *E. coli* isolates were conducted to investigate the dissemination patterns of *fosA3*-positive transposons and their links to different hosts and geographic regions.

## 2. Results

### 2.1. Higher Phenotypic Detection of Fosfomycin Resistance Compared to PCR Screening

Among the 400 *E. coli* isolates analyzed, 75 (19%) displayed phenotypic resistance to fosfomycin (growth at ≥256 µg/mL). The frequency of resistant isolates varied across the four integration farms, ranging from 8% to 35% (Table 1).

Of the 75 phenotypically resistant isolates, PCR screening identified the *fosA3* gene in 21% (16/75), corresponding to 4% (16/400) of all tested isolates. Notably, farm integration 2, which reported no recent history of fosfomycin use, exhibited the highest *fosA3* positivity rate (60%; 9/15). No isolates carried the *fosA* or *fosC2* genes (Table 2).

### 2.2. Genetic Diversity and Resistance Profiles in fosA3-Positive Isolates

Whole-genome sequencing of seven *fosA3*-positive isolates revealed different clonal groups and plasmid replicon types. Four isolates belonged to phylogroup B1, associated with low virulence lineages. The remaining three isolates belonged to phylogroups A (ST10), B2 (ST131), and D (ST38), which are more frequently linked to extra-intestinal pathogenic *E. coli* (ExPEC) (Table S1). Resistance markers were widely distributed across the isolates, with all but one harboring *bla*_CTX-M-55_ on the same plasmid carrying *fosA3*. Additional resistance determinants included AmpC β-lactamases (*bla*_CMY-2_), mutations conferring fluoroquinolone resistance (*gyrA*, *parC*), and aminoglycoside resistance genes. These findings particularly highlight the co-selection potential of *fosA3* and ESBL-associated resistance on transmissible plasmids (Table S1).

### 2.3. Association of fosA3 and ESBL Genes with Host and Region

A comparative analysis of 133,541 publicly available *E. coli* genomes revealed that 35% harbored transposon structures similar to those found in the Brazilian isolates (≥80% nucleotide identity). Among these genomes, *fosA3* was detected in 2% (973/46,410), with co-occurrence of *bla*_CTX-M-55_ in 45% of *fosA3*-positive isolates (441/973). Statistical analysis indicated significant associations between *bla*_CTX-M-55_ and specific isolation sources (i.e., human, animal, or environmental origin) (*p* = 0.05) with environmental isolates having the highest prevalence of *bla*_CTX-M-55_. The geographical origin was also significantly associated with ESBL resistance (*p* < 0.001), featuring higher *bla*_CTX-M-55_ carriage in South American isolates compared to those from Europe and the Middle East (Table S2). The highest estimated carriage probabilities were observed in South American and Asian isolates across all sources: humans (54% and 52%), animals (52% and 49%), and environments (62% and 60%, respectively) (Table S3). Logistic regression of Brazilian *E. coli* isolates (*n* = 444) further revealed that *fosA3* carriage was significantly associated with poultry (30%), followed by food (17%) and livestock (12%), compared to human isolates (2.5%) (Table S4). The likelihood of fosfomycin resistance was significantly higher in poultry isolates compared to other livestock sources (*p* = 0.03).

### 2.4. Shared Genetic Structures in fosA3-Positive Plasmids

**Figure 1:**
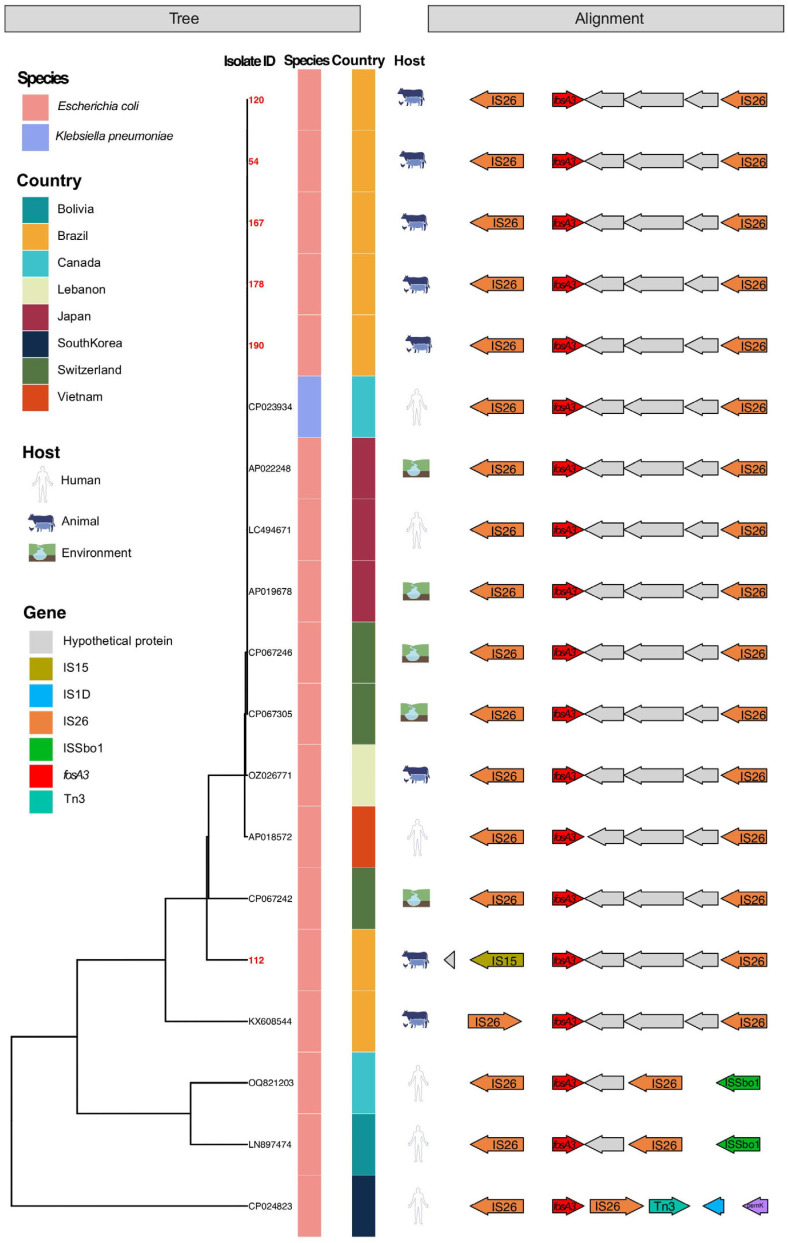
Schematic visualization of genetic structures flanking the *fosA3* gene among plasmids sharing > 90% nucleotide identity. Isolates are color-coded by host and region, with study isolates highlighted in red.

To assess the global dissemination of *fosA3*-positive plasmids, sequences with >90% nucleotide identity to Brazilian isolates were retrieved from the NCBI database. These plasmids originated from diverse hosts (human, livestock, and environment) and geographic regions, including major importers of Brazilian poultry meat (Table S5). Most sequences shared a conserved genetic structure, with *IS*6-family elements flanking the *fosA3* gene. Variations among the plasmids included differences in hypothetical proteins within the transposon, the orientation of *IS*26 transposase genes, and the presence of an *IS*15 element downstream of *fosA3* in one isolate (Figure 1). Despite these minor differences, the overall architecture of the composite transposon containing *fosA3* and *bla*_CTX-M-55_ remained conserved across hosts and regions.

**Figure 2:**
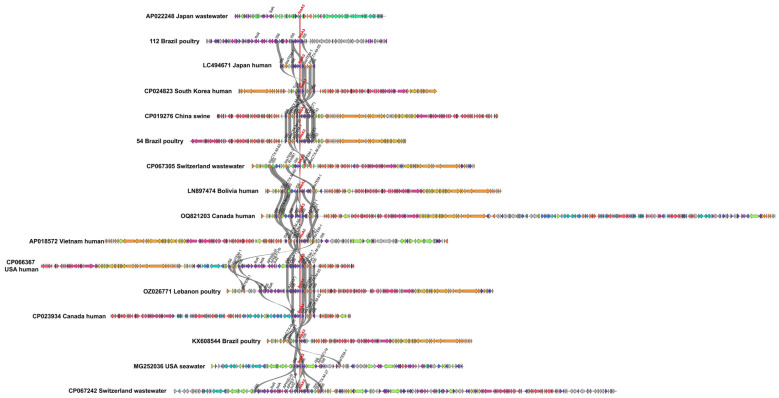
Alignment of complete plasmid structures carrying *fosA3*, highlighting shared transposon regions and adjacent resistance genes.

Whole-plasmid comparisons further confirmed the structural similarity of *fosA3*-positive plasmids from Brazilian poultry and international sources. *IS*26-flanked composite transposons were consistently observed, with adjacent resistance genes such as *bla*_TEM-1_ and *bla*_CTX-M-55_ commonly present. In one isolate (GenBank ID AP022248, Japan), the *fosA3* transposon lacked some structural components, indicating regional variability (Figure 2).

### 2.5. Phylogenetic Analysis of fosA3-Positive Isolates

**Figure 3:**
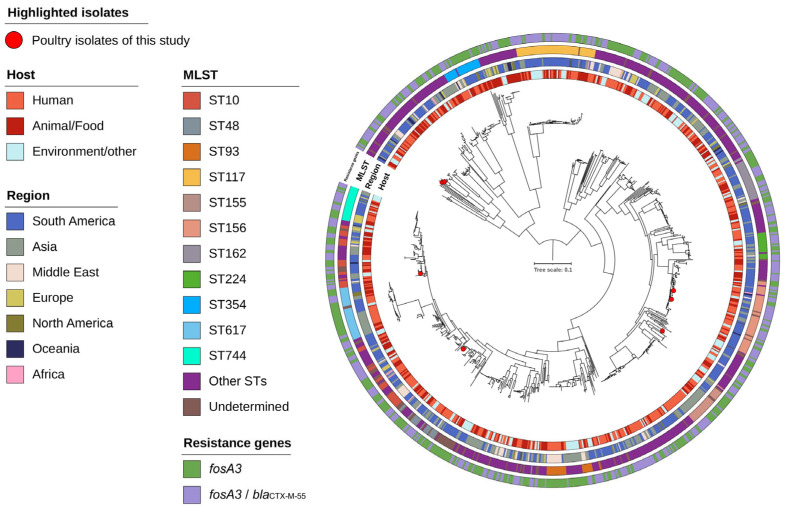
Mid-point rooted core genome phylogeny of 973 *fosA3*-positive *Escherichia coli* isolates. From the inner to the outer ring the tree features for each isolate are annotated by host, region, MLST, and AMR carriage, with Brazilian poultry isolates highlighted. The phylogeny was built on calling 39% (2.05 Mb) of the reference genome.

Core-genome phylogenetic analysis of 973 *E. coli* isolates with *fosA3* transposons (≥80% identity) was constructed using 39% (2.05 Mb) of the reference chromosome and showed no distinct clustering by host or geographic origin. Brazilian poultry isolates were dispersed across the phylogenetic tree, intermingling with human, animal, and environmental isolates from various regions (Figure 3). Most *fosA3*-positive isolates were of South American and Asian origin (519/973 and 301/973, respectively).

**Figure 4:**
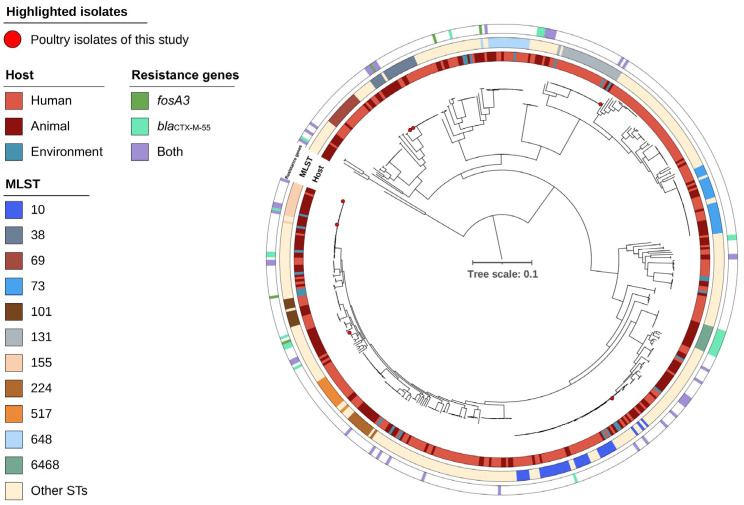
Mid-point rooted core-genome phylogeny of 444 Brazilian *Escherichia coli* isolates, illustrating intermingling of host sources and variation in AMR gene profiles. The poultry isolates of this study are marked with a red circle at the tip of the branch. The phylogeny was built on calling 34% (1.86 Mb) of the reference genome.

A separate phylogenetic analysis of Brazilian genomes (*n* = 444) demonstrated similar intermixing of human, animal, and environmental isolates, calling SNPs in 34% (1.86 Mb) of the reference genome. Some human isolates clustered closely with poultry isolates from this study; however, they lacked the *fosA3* and *bla*_CTX-M-55_ co-carriage typical of poultry isolates (Figure 4).

## 3. Discussion

This study reports an overall frequency of phenotypical fosfomycin resistance of up to 19% present in Brazilian broiler farms fecal isolates, however only 4% were PCR-positive and solely for the *fosA3* gene encoding enzymatic resistance. Long-read sequencing of seven of these isolates confirmed the *fosA3* presence on Inc-type plasmids, of which, most (6/7) co-carried ESBL resistance (*bla*_CTX-M-55_). These genes were located in a composite transposon that is found worldwide. The host variable had a significant impact on the chance of carrying ESBL among *fosA3*-positive isolates. South American isolates exhibited significant co-carriage of fosfomycin and ESBL resistance markers, as well as higher chances of being distributed among all tested host sources (humans, animals, environment) when analyzing both the host and region simultaneously. Of note, both phylogenies showed no exclusivity of hosts or geographical areas of origin, highlighting an apparent unrestricted horizontal dissemination of resistance markers against these high-priority antibiotic classes.

The sequenced isolates displayed different STs, serotypes and phylogroups among the investigated farms, indicating that the fosfomycin resistance was horizontally transmitted. Though some isolates (IDs 97 and 190; 112 and 167, Table S1), presented the same ST, serotype and phylogroup, all displayed carriage of additional assorted plasmids (Table S1). This could indicate an original common source for some of the circulating strains in addition to the horizontal acquisition of *fosA3* independently of clonal dissemination.

Phylogrouping showed that the isolates of this study, of which several belonged to lineages not frequently considered pathogenic such as A and B1, carried relatively few virulence factor profiles. This implies that most are part of the intestinal microbiota but nevertheless have acquired resistance plasmids in order to persist in this environment, linking the history of antibiotic use on these farms with the presence of resistant strains in the intestinal microbiota (Table S1). However, the potential for more virulent strains is also present, highlighted by isolates 54 (ST131) and 178 (ST10), carrying virulence factors linked to invasion (*ibeA*), iron acquisition (*fyu*, *iro*, *iuc*, *iut*, *sit*), capsule formation (*kps*, *neu*), and immune system evasion (*iss*, *omp*T) (Table S1).

The frequency of *fosA* carriage varies in different studies, given the variable local conditions and making direct comparisons challenging. For instance, in Brazil studies have identified varying frequencies whether in meat, cloacal swabs, and litter from healthy poultry as low as 0.7% [6] and as high as 40% [7]. The *fosA3* allele is the most frequently detected fosfomycin-modifying enzyme worldwide causing resistance [5] and its dissemination has been closely associated with different *bla*_CTX-M_ types [3].

Here, the *fosA3* was detected at a low frequency (4%), compared to the overall phenotypic resistance (19%), showing the presence of *fosA3* plasmid-mediated resistance and suggesting other resistance mechanisms for the remaining isolates. Studies worldwide have reported these findings which are likely related to chromosomal mutations such as those observed in the *murA* target gene or in the transporter genes, *glpT*, *uhpT*, though final confirmation is rarely pursued [18,19,20]. It is worth mentioning that the CLSI guidelines were followed for interpretation of the phenotypic results, and the number of identified phenotypic resistant isolates (or identifying those that are intermediate) could have been higher using different thresholds such as those by EUCAST; however, these were not used in this study. Phenotypic fosfomycin resistance in the absence of the usually screened resistance genes is common, implying that the observed resistance is likely of chromosomal origin [7,13,17].

The phylogenetic relatedness of isolates carrying fosfomycin resistance showed an intermingling between different hosts and regions, indicating extensive horizontal transmission of this resistance marker regardless of the two variables (Figure 3). Similarly, a study evaluating *E. coli* colistin-resistant isolates carrying the *mcr-1* gene also reported no clear clustering based on host or geographic origins, illustrating how plasmids driving widespread horizontal resistance can be detected worldwide [21].

However, a statistically significant difference in *bla*_CTX-M-55_ gene carriage between sources was observed among *fosA3*-positive isolates in the international datasets, with a higher prevalence for environmental isolates. South American fosfomycin-resistant isolates were significantly more ESLB-resistant regardless of source. This suggests that the widespread use of these antibiotics in this region is not only dispersed by livestock but also by human medical use with both sources likely contributing to environmental dispersal. Among the Brazilian isolates, the results were similar except that more animal-related isolates carried either *fosA3*, *bla*_CTX-M-55,_ or both (Figure 4). Further statistical analyses were not performed due to the fewer numbers of Brazilian isolates. However, the probability of carrying *fosA3* in Brazilian livestock was higher compared to humans, and poultry was estimated to carry more *fosA3* resistance (30%) compared to other livestock (12%) (Table S4).

Studies in other parts of the world have reported increasing fosfomycin resistance in animal species, particularly in poultry [22,23]. In China, frequencies of up to 7% in fecal isolates from healthy chickens were described [11]; however, the numbers increase considerably in studies that are not strictly focused on fosfomycin but also report ESBL-producing isolates given the common co-location of fosfomycin and ESBL resistance on the same plasmids [12,13].

It is reported that the detection of *fosA3* is strongly associated with *bla*_CTX-M_ groups in retail poultry meat and asymptomatic individuals in Brazil [6,7]. Here, all seven isolates but one carried *bla*_CTX-M-55_ in addition to *fosA3*, in addition to other AMR genes (Table S1).

Fosfomycin is not widely used worldwide in animal production. However, several countries in Latin America use it as a therapeutical agent in poultry and swine for controlling specific multi-resistant bacterial infections [1]. Fortunately, overall resistance among human clinical isolates is mostly low, thus, treating MDR *E. coli* infections with fosfomycin is common [2,24]. Conversely, a 50% increase in clinical use of fosfomycin caused increased resistance rates among human isolates in Spain [16,25] and in Israel [15].

The mostly low resistance rates observed in the clinical practice are related to the fitness cost of harboring resistance genes when considering chromosomal mutation mechanisms [4]. Still, the fitness cost of fosfomycin-modifying enzymes, especially the *fosA3* gene, is lower as it is frequently plasmid-encoded, facilitating the dissemination among *E. coli* and other *Enterobacterales* whether in the animal or human microbiota [3]. High rates of *fosA3* in Chinese poultry and other animals have been reported, but a low prevalence has been detected among humans in Europe and North America. This suggests that the clinical cases in humans in Western countries have their background in imported food sources or travelling to and from Asia [16]. Therefore, even if the current resistance in human isolates is low, this could change with the spread of plasmid-mediated *fosA* genes, and increased fosfomycin use to treat human disease, as well as a co-selection via other antibiotics [16]. Conversely, *fosA* and *fosC2* genes were not detected, which has also been the case for most studies where these variants are detected in a lesser proportion compared to *fosA3* or are absent [13,26].

Independently of the chromosomal or plasmidial location, insertion sequences are frequently found flanking these resistance genes facilitating the mobilization and spreading [3]. The success of *fosA3* and its dissemination is linked to IS26 family sequences flanking this gene, facilitating the transposition as well as association with highly self-transferrable IncFII replicons frequently containing co-resistance [27,28,29]. Most *fosA3* positive isolates in this study carried IncFII plasmids (Table S1).

Here, the *fosA3* gene was shown to be part of a transposon, and sequences sharing a higher identity with this transposon were present in 46,410 (35%) of all available *E. coli* isolates worldwide on EnteroBase. The statistical analyses with the highest identity isolates featuring both the transposon and *fosA3* (N = 973), were not able to identify links to particular hosts though the region is significant for resistance. Most isolates had the known type A composite transposon configuration with *IS*26 family sequences flanking the *fosA3*, *bla*_CTX-M-55_ and *bla*_TEM-1_ genes (Figure 2) which is reported worldwide [29], particularly in Asia, where *fosA3* is disseminated [30,31]. However, the statistical analyses among international and Brazilian-only datasets were not comparable since the first group included only those that had a higher identity with our isolates and were positive for *fosA3*, while the Brazilian dataset included all available regardless of identity and *fosA3* carriage due to fewer numbers for comparisons. This bias could be addressed in future studies as additional sequences are released. Nonetheless, the current results do suggest an important correlation among South American *fosA3* resistance, its usage in this region and dissemination among different sources.

The use of ceftiofur injection *in ovo* at hatcheries is permitted in Brazil and is known to contribute to the selection and shedding of ESBL *E. coli* in one-day-old chickens [32]. Therefore, third-generation cephalosporin use can also drive resistance through co-selection of plasmids containing ESBL, even in the absence of fosfomycin administration. In Chinese poultry farms, where fosfomycin is officially not allowed, high resistance has been linked to co-selection by other antimicrobials [13].

The therapeutical use of fosfomycin is allowed in Brazilian poultry farms and in this study, all but one integration farm reported using fosfomycin. This resistance could be linked to the selective pressure of fosfomycin administration, but a ceftiofur co-selection and dissemination through plasmids co-harboring resistance is likely contributing to this situation given the high fosfomycin treatment costs in Brazil. The latest hypothesis could relate to the group of farms belonging to the same integration (number 2) where no recent use of fosfomycin was reported, while conversely showing the highest *fosA3* carriage rate (60%) (Table 2). This integration is the only one that did not have its own matrices and acquired them from facilities that could have used ceftiofur. Similarly, ref. [17] reported one-day-old chicks exported from Brazil to Uruguay to carry *fosA3* and *bla*_CTX-M-55_ in *E. coli* fecal isolates. Since fosfomycin use is forbidden in poultry in Uruguay, the resistance was linked to Brazil.

One important limitation among most antimicrobial surveillance agencies around the world is that determining fosfomycin resistance is not a routine test, though this should be an integral part of developing a control strategy worldwide for this drug [3]. Since Brazil is a significant meat producer and exporter, the use of antibiotics in the country also has international consequences [9,33]. This is of significance as fosfomycin remains one of the last and best options to treat infections caused by MDR and XDR (extensively drug-resistant) bacteria [1]. Such isolates have been increasingly reported, as in the case of varied *Enterobacterales* co-carrying ESBL, *fosA3*/*mcr* in the clinical and veterinary settings or carrying *fosA3*/*mcr*/NDM-1/KPC-2 both in humans and in livestock, including poultry [29], thus, posing severe obstacles to the successful treatment of human clinical cases.

## 4. Materials and Methods

### 4.1. Selection of Production Systems

This study was conducted in 2020 across four poultry integration systems in Southeastern Brazil. A summarized workflow describes the methods used in this study (Supplementary Figure S1). These systems comprised medium-sized broiler operations with slaughter capacities ranging from 100,000 to 150,000 birds per day. Within each integration system, 20 poultry houses were randomly selected, and five individual fecal samples were collected per house, resulting in a total of 400 samples. Fecal samples were collected from freshly voided droppings using sterile swabs and immediately placed into a Cary–Blair transport medium (Laborclin, Paraná, Brazil). Samples were kept at 4 °C during transport and processed within 24 h. Three of the four integrations reported using fosfomycin as an oral therapeutic agent within the six months prior to sampling, while one system reported no recent usage.

### 4.2. Bacterial Isolation and Identification

The swabs containing fecal samples were inoculated into brain-heart infusion (BHI) (BD DIFCO, Franklin Lakes, NJ, USA) broth at 37 °C for 18 h. Enrichment cultures were streaked onto MacConkey agar (BD DIFCO) and incubated at 37 °C for 24 h. All 400 samples yielded lactose-fermenting colonies presumptively identified as *Escherichia coli* and one predominant colony per sample was selected, subcultured to confirm it as a single colony, and confirmed as *E. coli* using matrix-assisted laser desorption/ionization-time of flight mass spectrometry (MALDI-TOF MS) on a Microflex LRF system (Bruker Daltonik, Bremen, Germany). *E. coli* isolates were stored in Luria-Bertani (LB) (BD DIFCO) broth supplemented with 20% glycerol at −80 °C for further analysis.

### 4.3. Evaluation of the Resistance Phenotype

Fosfomycin resistance was determined using the agar dilution method following Clinical and Laboratory Standards Institute (CLSI) guidelines. Mueller–Hinton agar (BD DIFCO) supplemented with 1.5% glucose-6-phosphate was prepared with a final fosfomycin concentration of 256 µg/mL. Lower concentrations were not used given that at the time of the experiments, only the CLSI was officially recognized for fosfomycin evaluation and therefore, no intermediate resistance was detected. Bacterial suspensions were adjusted to a 0.5 McFarland standard and inoculated onto the agar plates. Plates were incubated at 37 °C for 24 h. Isolates exhibiting growth at or above the cutoff concentration [34] were classified as fosfomycin-resistant. *E. coli* ATCC 25922 served as a quality control strain.

### 4.4. Genotypic Characterization

Fosfomycin-resistant isolates had the DNA extracted with a boiling method [35] and screened using a triplex PCR for the presence of *fosA*, *fosA3*, and *fosC2* genes. Briefly, the reagents consisted of 10 mM Tris-HCl (ph 8.3), 50 mM KCl, 1.5 MgCl_2_, 200 μM dNTP mix, each primer pair, 0.5 U Taq polymerase (Invitrogen, Carlsbad, CA, USA) and 50 ng of the template DNA, in 30 cycles of 1 min at 94 °C, 1 min at 58 °C, and 2 min at 72 °C, with a final extension of 72 °C for 8 min [18]. Positive controls previously obtained from clinical isolates present in the collection of the School of Veterinary Medicine and Animal Science (Universidade de São Paulo) and confirmed to carry the genes were used, while *E. coli* MG1655 (ATCC 700926) was used as a negative control. Amplicons were visualized on 1.5% agarose gels stained with SYBR Safe DNA Gel Stain (Thermo Fisher Scientific, Waltham, MA, USA).

### 4.5. Whole-Genome Sequencing and Plasmid Analysis

Seven *fosA3*-positive isolates were selected for whole-genome sequencing (WGS) using Oxford Nanopore Technologies (ONT). Single colonies from fresh blood agar cultures were resuspended in enzymatic lysis buffer containing Proteinase K, Triton X-100, Tris-HCl, EDTA (Thermo Fisher Scientific, Waltham, MA, USA), and phosphate-buffered saline. DNA was extracted using the automated MagLEAD 12 gC system and quantified using the Qubit Broad Range dsDNA assay, using 20 ng/µL of the extracted DNA for the reaction.

Sequencing libraries were prepared with the Rapid Barcoding Kit (SQK-RBK114.96, Oxford Nanopore Technologies, Oxford, UK) and sequenced on MinION R10.4.1 flow cells using a GridION platform. Basecalling was performed with Guppy software v6.6.0., and high-quality reads were assembled using Flye v2.9. with the --nano-hq option. Annotated contigs were visualized using Bandage v0.8.1 [36] and the extracted circularized sequences containing each plasmid were annotated with Bakta v1.7 [37] using the standard settings.

Antimicrobial resistance (AMR) genes were identified using the abriTAMR pipeline [38] with the standard built-in software parameters. Additional genomic features were typed for clonal lineages with MLST v2.16 (similarity cutoff >90%), phylogroups with EzClermont (https://ezclermont.hutton.ac.uk (accessed on 15 July 2024)) (standard settings), and serotypes with ABRicate v0.9.0 (https://github.com/tseemann/abricate (accessed on 15 July 2024)) using the EcOH tool (>90% for nucleotide similarity and coverage) [39,40,41]. Virulence factors were identified with AMRFinderPlus v3.12.8 using abriTAMR and standard settings, and FimH typing was performed with FimTyper v1.0 (>95% nucleotide similarity) [42].

### 4.6. Global Comparative Genomic Analysis

To evaluate the global dissemination of the *fosA3*-containing transposon, a dataset of *E. coli* genomes (*n* = 133,541) was retrieved from EnteroBase (October 2022). These genomes were screened for sequences sharing >80% nucleotide identity with the transposon identified in this study. Isolates positive for *fosA3* and containing the transposon region (4 Kb) were included in further phylogenetic and statistical analyses.

For a comparative analysis, plasmid sequences with >90% nucleotide identity to the *fosA3* transposon were retrieved from the NCBI database using BLASTn v2.16.0. The genetic regions surrounding *fosA3* and associated resistance genes were analyzed using clinker v0.0.27 [43] for the whole-plasmid alignment, while the flanking regions surrounding *fosA3* with Flankophile v0.2.10 [44].

### 4.7. Phylogenetic Analysis

Core-genome single nucleotide polymorphism (SNP)-based phylogenetic analysis was performed to investigate the relationships among *fosA3*-positive isolates. Seven sequenced isolates from this study, along with *fosA3*-positive international isolates from human, animal, and environmental sources (*n* = 966), were included. SNP calling was conducted using the NASP v1.0.0 pipeline with GATK v4.2.2, using the ASM433178 genome as a reference [45]. Phylogenetic trees were constructed with IQ-TREE v2.1.2 using ModelFinder and 100 bootstrap replicates. Visualization was performed using iTOL v6.7.6 (https://itol.embl.de/).

For comparative analysis specific to Brazil, all *E. coli* genomes with metadata available in EnteroBase at that time (*n* = 444) were included, regardless of *fosA3* carriage or transposon presence. This allowed for a broader understanding of the relationships between Brazilian isolates and those from international sources.

### 4.8. Statistical Analysis

Statistical analyses were performed separately for Brazilian and global datasets. For Brazilian isolates, logistic regression was used to assess associations between host sources (poultry, livestock, food, humans) and *fosA3* carriage. For the global dataset, multivariate logistic regression was conducted to evaluate the influence of host source and geographic region (with South America as the reference) on the likelihood of *fosA3* and *blaCTX-M-55* co-carriage. A Wald test was used to determine the significance of variables in the models. Isolates from Oceania and Africa were excluded from these analyses due to insufficient sample sizes (<17 and <7 isolates, respectively). Statistical analyses were performed using R v4.2.1 [46].

## 5. Conclusions

This study shows the presence of fosfomycin-resistant isolates in fecal samples of healthy broilers in Southeastern Brazil, an important source for meat production both for national and international markets. The presence of antimicrobial resistance on highly transmissible plasmids associated with well-known composite transposons co-carrying ESBL genes is linked to widespread fosfomycin resistance in different hosts and environments worldwide. This reinforces the need for a surveillance system and better use of this drug in Brazilian livestock as well as those involved in co-selection mechanisms (especially third-generation cephalosporins). Realistic alternative treatments and preventative measures in the poultry sector are of high importance given this drug is of vital use in human medicine.

## Figures and Tables

**Table 1:** Phenotypic results for 400 *Escherichia coli* isolates tested for resistance against fosfomycin via the agar dilution technique according to each sampling integration farm.

Integration Farm	Susceptible	Resistant
Integration 1 (N = 100)	67%	33%
Integration 2 (N = 100)	85%	15%
Integration 3 (N = 100)	92%	8%
Integration 4 (N = 100)	80%	20%

**Table 2:** PCR results for the amplification of the *fosA3* gene in *Escherichia coli* isolates phenotypically resistant to fosfomycin on each sampled farm.

Integration Farms	Phenotypically Resistant Isolates	Positive isolates		
		*fosA3* Positive Isolates	Of the Total Phenotypically Resistant Isolates	Of the Total Isolates (N = 400)
Integration 1	33	2	6.06%	0.5%
Integration 2	15	9	60.00%	2.25%
Integration 3	8	3	37.50%	0.75%
Integration 4	19	2	10.53%	0.5%
Total	75	16	-	-

## Data Availability

The sequenced data is available online at the National Center for Biotechnology Information—NCBI (bioproject PRJNA715669).

## Supplementary Materials

The following supporting information can be downloaded at: https://www.mdpi.com/article/10.3390/antibiotics14030269/s1, Figure S1: Summarized flowchart describing the collection and processing of isolates. A: Four different integration farms were sampled, yielding 400 fecal samples; B: *Escherichia coli* colonies were isolated and resistant strains were selected with the agar dilution method; C: DNA was extracted and tested with specific primers for *fosA/A3/C* genes; D: Selected PCR positive isolates were long-read sequenced and the sequences used for further analyzes.; Table S1: *In silico* typing details for each sequenced Brazilian poultry isolate.; Table S2: Statistical analyses for the presence of ESBL among host and region of origin in the international dataset of *fosA*-positive *E. coli* isolates.; Table S3: Estimated probabilities (%) of *bla*_CTX-M_ carriage among Fosfomycin-resistant *E. coli.*; Table S4: Percentage of estimated probability of *fosA* carriage among different sources of Brazilian isolates. Table S5: Selected complete plasmid sequences from NCBI used for comparisons of the flanking regions of the *fosA3* gene.
