# Supplementary material for: Escherichia coli in Brazilian Poultry Fecal Samples: Co-Carriage of Fosfomycin and ESBL Resistance
Source: Antibiotics (Basel). 2025 Mar 6;14(3):269. doi: 10.3390/antibiotics14030269 (PMC11939591; doi:10.3390/antibiotics14030269)
Supplement: Supplementary file 1 [file antibiotics-14-00269-s001.zip › Suppl. Table S2.pdf]

**Supplementary Table S2.** Statistical analyses for the presence of ESBL among host and region of origin in the international dataset of *fosA3* positive *E. coli* isolates. The "Human" and "South America" variables were set as the references for the "Source" and "Geographical origin", respectively.

| Variables | OR (95%CI) | Logistic regression p-value | Wald test p-value |
|---|---|---|---|
| **Source** | | | 0.05 |
| Human | 1 | | |
| Animal | 0.9 (0.7-1.2) | 0.5 | |
| Environment | 1.4 (1.0-2.0) | 0.07 | |
| **Geographical origin** | | | <0.001 |
| South America | 1 | | |
| North America | 0.8 (0.3-2.0) | 0.6 | |
| Europe | 0.2 (0.1-0.4) | <0.001 | |
| Middle East | 0.2 (0.1-0.3) | <0.001 | |
| Asia | 0.9 (0.7-1.2) | 0.5 | |
