# Supplementary material for: Escherichia coli in Brazilian Poultry Fecal Samples: Co-Carriage of Fosfomycin and ESBL Resistance
Source: Antibiotics (Basel). 2025 Mar 6;14(3):269. doi: 10.3390/antibiotics14030269 (PMC11939591; doi:10.3390/antibiotics14030269)
Supplement: Supplementary file 1 [file antibiotics-14-00269-s001.zip › Suppl. Table S3.pdf]

Supplementary Table S3. Estimated probabilities (%) of *bla*$_{CTX-M}$ carriage among fosfomycin resistant *E. coli.*

Host	Continent	Estimated probabilities (%) of *bla*$_{CTX-M}$ carriage among fosfomycin resistant *E. coli*
Human	South America	54.1
Human	North America	48.2
Human	Europe	17.6
Human	Middle east	17.8
Human	Asia	51.7
Animal	South America	51.8
Animal	North America	45.9
Animal	Europe	16.3
Animal	Middle east	16.5
Animal	Asia	49.4
Environment	South America	62.3
Environment	North America	56.6
Environment	Europe	23.1
Environment	Middle east	23.3
Environment	Asia	60.1
