# Supplementary material for: Escherichia coli in Brazilian Poultry Fecal Samples: Co-Carriage of Fosfomycin and ESBL Resistance
Source: Antibiotics (Basel). 2025 Mar 6;14(3):269. doi: 10.3390/antibiotics14030269 (PMC11939591; doi:10.3390/antibiotics14030269)
Supplement: Supplementary file 1 [file antibiotics-14-00269-s001.zip › Suppl. Table S4.pdf]

Supplementary Table S4. Percentage of estimated carriage of *fosA3* carriage among different sources of Brazilian isolates.

**Host**	**Estimated (%)**
Human	2.5
Poultry	29.6
Livestock	11.6
Other animals	12.5
Food	16.7
Environment	7.7
