# Supplementary material for: Escherichia coli in Brazilian Poultry Fecal Samples: Co-Carriage of Fosfomycin and ESBL Resistance
Source: Antibiotics (Basel). 2025 Mar 6;14(3):269. doi: 10.3390/antibiotics14030269 (PMC11939591; doi:10.3390/antibiotics14030269)
Supplement: Supplementary file 1 [file antibiotics-14-00269-s001.zip › Suppl. Table S5.pdf]

Supplementary Table S5. Selected complete plasmid sequences from NCBI used for comparisons of the flanking regions of the *fosA3* gene.

ID	Country	Host	Species
**CP023934.1**	Canada	*Homo sapiens*	*Klebsiella pneumoniæ*
**AP022248.1**	Japan	wastewater	*Escherichia coli*
**LC494671.1**	Japan	*Homo sapiens*	*Escherichia coli*
**AP019678.1**	Japan	wastewater	*Escherichia coli*
**CP067246.1**	Switzerland	water	*Escherichia coli*
**CP067305.1**	Switzerland	wastewater	*Escherichia coli*
**OZ026771.1**	Lebanon	poultry	*Escherichia coli*
**AP018572.2**	Viet Nam	*Homo sapiens*	*Escherichia coli*
**CP067242.1**	Switzerland	water	*Escherichia coli*
**KX608544.1**	Brazil	poultry	*Escherichia coli*
**OQ821203.1**	Canada	*Homo sapiens*	*Escherichia coli*
**LN897474.2**	Bolivia	*Homo sapiens*	*Escherichia coli*
**CP024823.1**	South Korea	*Homo sapiens*	*Escherichia coli*
