# Supplementary figures and images for: Escherichia coli in Brazilian Poultry Fecal Samples: Co-Carriage of Fosfomycin and ESBL Resistance
Source: Antibiotics (Basel). 2025 Mar 6;14(3):269. doi: 10.3390/antibiotics14030269 (PMC11939591; doi:10.3390/antibiotics14030269)

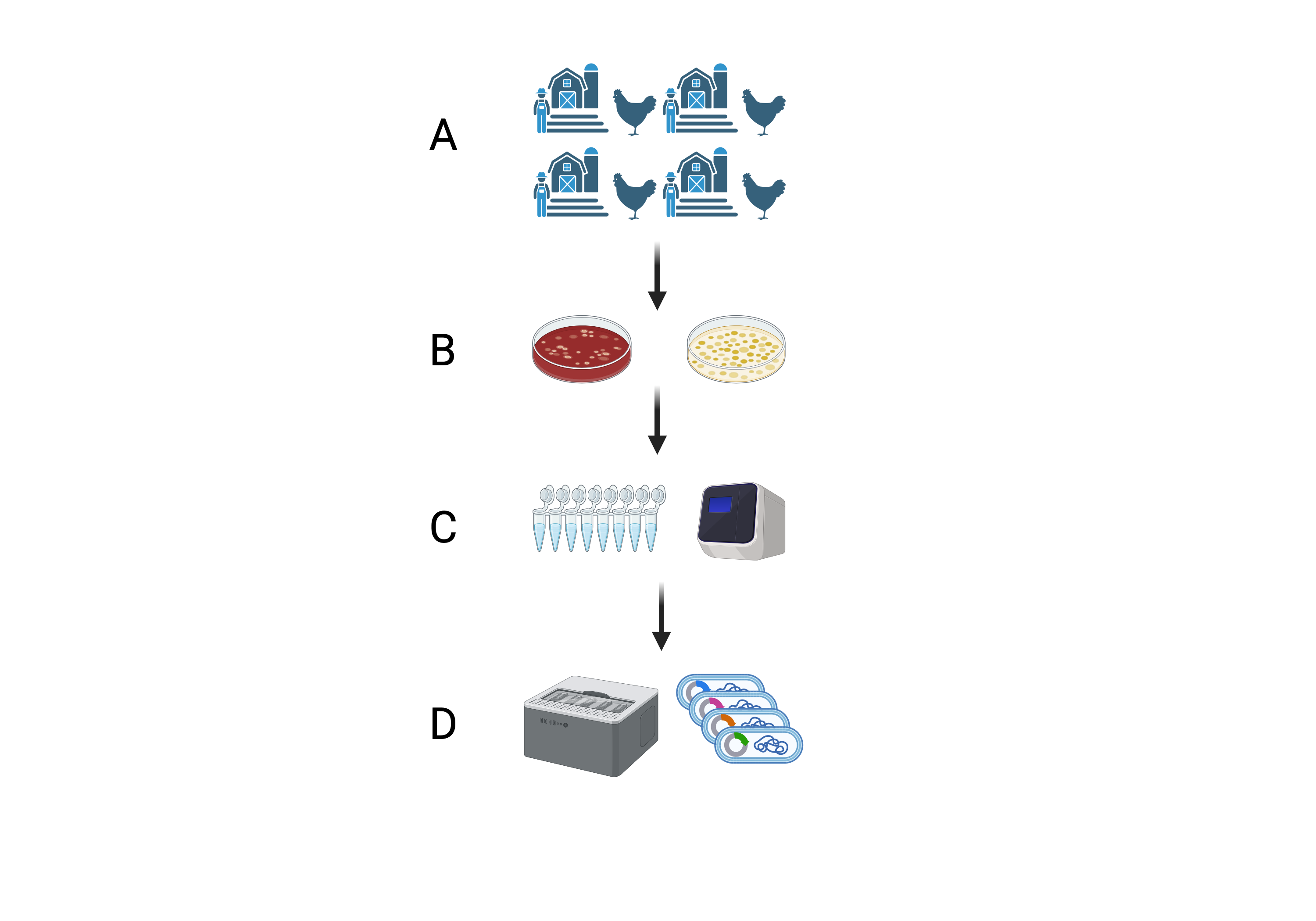

Supplement: Supplementary file 1 [file antibiotics-14-00269-s001.zip › Suppl. Figure S1.png]
